# Optoelectronic properties and device simulation of ZnS polymorphs as buffer layers for CZTSSe solar cells

**DOI:** 10.1039/d5ra07195j

**Published:** 2025-11-18

**Authors:** Md. Azad Patwary, Aqib Adnan Shafin, Md. Morshed Alam, Rajat Kumar Singh Durjoy, Norasikin Ahmad Ludin, Mohd Sukor Su'ait, Md. Akhtaruzzaman, M. Mottakin

**Affiliations:** a Department of Applied Chemistry and Chemical Engineering, Gopalganj Science and Technology University Gopalganj-8105 Bangladesh mottakin@gstu.edu.bd; b Solar Energy Research Institute, Universiti Kebangsaan Malaysia Bangi Selangor Darul Ehsan 43600 Malaysia sheekeen@ukm.edu.my; c Department of Chemical Engineering, Faculty of Engineering, Islamic University of Madinah M adinah 42351 Saudi Arabia makhtar@iu.edu.sa

## Abstract

Kesterite (CZTSSe) has emerged as a sustainable thin-film absorber, yet its device efficiencies remain below those of leading photovoltaic technologies. Optimizing the buffer layer (BL) is a promising strategy to overcome these limitations. Here, we combined density functional theory (DFT) calculations with SCAPS-1D simulations to systematically evaluate ZnS polymorphs (cubic, hexagonal, trigonal) as a BL for CZTSSe solar cells. DFT analysis (GGA-PBE, CASTEP) reveals band gaps of 3.51 eV (cubic), 3.52 eV (hexagonal), and 3.53 eV (trigonal). The hexagonal phase exhibits superior carrier transport properties with electron and hole mobilities of 343.2 and 92.6 cm^2^ V^−1^ s^−1^, respectively. Density-of-states analysis confirms Zn-3d orbitals lie deep in the valence band, with S-3p levels predominating close to the Fermi level, and Zn-4s/4p defining the conduction band, highlighting S-3p → Zn-4s/4p transitions. SCAPS-1D simulations for the device ITO/AZO/ZnS/CZTSSe/Au demonstrate that the crystal phase of ZnS strongly impacts photovoltaic performance. Utilizing hexagonal ZnS BL achieves the highest efficiency (PCE 14.18%, *J*_SC_ 25.93 mA cm^−2^, FF 62.5%) due to the higher mobility of that crystal system. Furthermore, systematic variation of ZnS thickness, donor density, mobility, band gap, and bulk/interface defect densities, along with back-contact work function and operating temperature, reveals critical design parameters governing charge recombination, series resistance, and interfacial quality to improve performance. This combined theoretical-simulation study highlights that hexagonal ZnS emerges as the most effective BL for CZTSSe solar cells, offering superior carrier transport and interfacial stability for enhanced device efficiency.

## Abstract 2

Kesterite (CZTSSe) is a sustainable thin-film absorber, but its efficiency still trails leading PV technologies. Studying ZnS polymorphs and using them as buffer layers can improve interface quality and significantly boost device performance.

## Graphical abstract

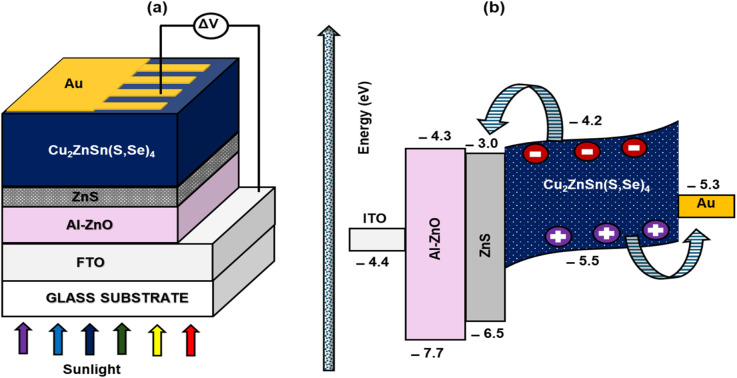

## 1 Introduction

To curb global warming, fossil fuels need to be substituted with renewable energy sources. Solar cells offer a viable solution to meet society's energy demands, as solar energy is abundant, clean, free, and environmentally friendly.^1^ While silicon dominates today's photovoltaic market, thin-film solar cell (TFSC) devices are gaining attention for their low cost, simple fabrication, eco-friendliness, and flexibility.^2–5^ Photons create pairs of electrons and holes in the absorbent layer in TFSCs, while the window layer allows light to enter and ensures proper band alignment with the front buffer layer (BL), thereby facilitating efficient charge collection.^6^ The commonly used CdS BL is toxic, In_2_S_3_ is a rare and costly material, while ZnS is eco-friendly with a direct wide band gap (3.2–3.9 eV), high broad-spectrum transmittance, superior optical characteristics, and environmentally friendly nature, making it a promising alternative.^7–10^ The ZnS BL makes it possible for photons with high energies to pass through the absorber layer, adjusting band alignment and also increasing the blue response to TFSCs.^11^ The stable crystals of zinc sulphide (ZnS) are hexagonal wurtzite (α-ZnS) and cubic zinc blende (β-ZnS).^12^ Notably, the wurtzite structure can undergo a distortion into a trigonal lattice, while the zinc blende structure may transform into a rhombohedral lattice.^13^ Such lattice distortions can occur during doping or under specific processing conditions.^14–16^

Kesterite-based materials, such as CZTSSe, are considered to be a potential photovoltaic substance for affordable, earth-abundant components, excellent optical energy band gap within 1.0 and 1.7 eV, along with an elevated absorption coefficient of >10^4^ cm^−1^.^17^ Previous studies have demonstrated that ZnS has been widely used as a BL in CZTSSe-based TFSC to enhance device performance. Felipe A. La Porta *et al.* worked DFT study on three different ZnS polymorphs; however, their relatively low band gaps rendered them unsuitable for application in TFSC.^18^ Mai Nguyen *et al.* investigated the application of ZnS BL for CZTSSe photovoltaic cells and reported that a thickness of 10–25 nm ZnS layer served as a highly effective BL, achieving a device efficiency of 4.50%.^19^ Ju Young Park *et al.* carried out an experiment on the ZnS BL layer in CZTSSe solar cells and found that an increment of 3.8% efficacy was attained using a selenium-rich absorber layer.^20^ Both studies show that the lack of efficiency of that type of solar cell is due to interface defects, non-optimal band alignment between the buffer and absorber layer. Therefore, there is a lot of room to increase the effectiveness of that kind of photovoltaic cells. S. Vallisree *et al.* worked on CZTS/ZnS/ZnO-based TFSC utilizing ZnS as the BL and reported an improvement in efficiency from 3.69% to 7.65% by adjusting thickness and defect states.^21^ Cu_2_ZnSnS_4_ thin-film photovoltaic cells with a ZnS BL along with ZnO:Al films were investigated by Lekhram Hirwani *et al.*; they achieved an optimised energy conversion rate of 32.99%, which is near the Shockley–Queisser limit.^22^ However, they did not explore CZTSSe solar cells, which motivates further study of CZTSSe devices with a thorough analysis of the ZnS BL. Literature shows a clear research gap in the detailed investigation of ZnS BL in CZTSSe-based TFSCs. Material modeling combined with device simulation is a powerful approach for analyzing the physical and chemical processes in TFSC, helping to identify defect mechanisms and guide strategies for improving efficiency.^23^ Combining CASTEP to study the optoelectronic properties of materials with SCAPS-1D for detailed analysis of ZnS polymorphs in TFSCs offers deeper insight to improve the performance of that type of solar cells.

In this study, to evaluate ZnS as a BL in the device structure of ITO/Al-ZnO/ZnS/CZTSSe/Au, we combined first-principles DFT calculations in CASTEP (Materials Studio) with one-dimensional SCAPS simulations. Initially, DFT will be used to study the optical, electronic, and structural properties of ZnS. Band arrangement, density of charges, and optical absorption are among the structural, electrical, and optical characteristics of ZnS polymorphs that will be examined using DFT. The performance of ZnS BL will then be examined using the SCAPS-1D. This hybrid approach allows precise assessment the suitability of ZnS as a BL and its impact on CZTSSe solar cell efficiency, providing insights for material selection and device design.

## 2 Computational methodology

### 2.1 DFT calculation

The optoelectronic properties of ZnS polymorphs were calculated using first-principles with the CASTEP code in Materials Studio 2024.^24^ Perdew–Burke–Ernzerhof, in combination with the generalized gradient approximation, was employed.^25^ DFT + U calculations were carried out with *U*_d_ = 8 eV applied to Zn-3d orbitals and *U*_p_ = 4 applied to S-3p states together with *U*_d_.^26^ In this work, an energy cutoff of 350.0 eV was applied to evaluate the exchange–correlation energy.^27^ A pseudopotential was used to characterise the ion-electron collisions, specifically the OTFG ultrasoft type. The Monkhorst–Pack scheme *k* points grid of 12 × 12 × 12 for cubic, 12 × 12 × 3 for hexagonal, and 12 × 12 × 2 for trigonal was used to simplify the Brillouin zone, and the relativistic treatment was Koelling–Harmon.^28^ The lattice parameters of ZnS after geometry optimization lengths and angles were *a* = *b* = *c* = 5.39 Å, *α* = *β* = *γ* = 90° for cubic, *a* = *b* = 3.81 Å and *c* = 12.45 Å, *α* = *β* = 90°, and *γ* = 120° for hexagonal, and *a* = *b* = 3.81 Å and *c* = 28.06 Å, *α* = *β* = 90°, and *γ* = 120° for trigonal, small deviation from the standard value, proving the feasibility to adopt this method for DFT calculations. For geometric optimization, the amount of energy, force, tolerance for stress, and displacement tolerances for convergence were established at 2.0 × 10^−3^ Å, 0.05 eV Å^−1^, 0.1 GPa, and 2 × 10^−5^ eV per atom, correspondingly.^29^ An overall energy variance of no more than 10^−5^ eV per atom is the threshold for geometry optimisation tolerances.^30^ Reliable structural configurations were guaranteed by these parameters, and the electrical band structure, DOS, and optical characteristics were then examined.^31^

### 2.2 SCAPS-1D simulation

**Fig. 1:**
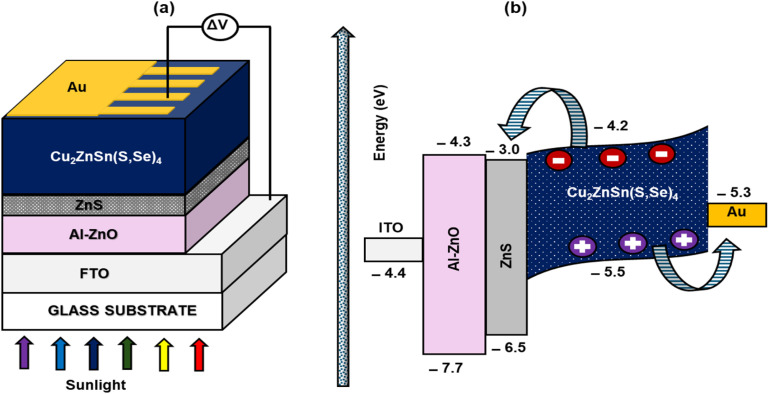
(a) Design of the proposed PSCs' device and (b) their energy band level.

The University of Gents' SCAPS-1D program primarily solves three crucial semiconductor equations—the continuity equation, the Poisson equation, and the charge transport equation for electrons and holes—to assess various solar cell properties.^32^ It allows researchers to model solar cells with up to seven layers through its cell definition panel, making it highly versatile for device simulation. While the computer program allows for precise management of both front and rear contacts and the assessment of physical characteristics, the control interface allows for the setting of conditions for operation. Calculations of short-circuit current density (*J*_SC_), voltage in the open circuit (*V*_OC_), fill factor (FF), conversion efficiency of power (*η*), quantum efficiency (QE), spectrum responses, and carrier production/recombination features are just a few of the many AC and DC electrical studies that SCAPS-1D is capable of doing.^33^ In contrast to a lot of other simulations, it provides a broader set of features for studying solar cell performance. Three solar cell architectures have been developed and tested in this study, and the band alignment of ITO/Al-ZnO/ZnS/CZTSSe/Au TFSC are shown in Fig. 1. Additionally, the simulation's optimised material characteristics are drawn from related research and also from DFT calculations and listed in Table 1. Here, the simulation is carried out for air mass 1.5G radiation (1000 W m^−2^, 300 K). The electron and hole velocity is 10^7^ ms^−1^.

**Table 1:** Parameters used in this simulation

Parameters	ITO	Al-ZnO^34^	ZnS^35^	CZTSSe^36^
Thickness (nm)	200	50	50	1000
*E*_g_ (eV)	3.6	3.37	3.52 (DFT)	1.3
*χ* (eV)	4.1	4.5	4.1	4.2
*ε*_r_	10	9	10	9.1
*N*_c_ (cm^−3^)	2.2 × 10^18^	2.22 × 10^18^	1.8 × 10^18^	2.2 × 10^18^
*N*_v_ (cm^−3^)	1.8 × 10^19^	1.9 × 10^19^	1.8 × 10^19^	1.9 × 10^19^
*µ*_e_ (cm^2^ V^−1^ s^−1^)	100	100	350 (DFT)	100
*µ*_h_ (cm^2^ V^−1^ s^−1^)	25	25	94.4 (DFT)	25
*N*_A_ (cm^−3^)	0	0	0	0
N_D_ (cm^−3^)	1 × 10^17^	1 × 10^18^	1 × 10^17^	1 × 10^18^

## 3 Result and discussion

### 3.1 Band structure

**Fig. 2:**
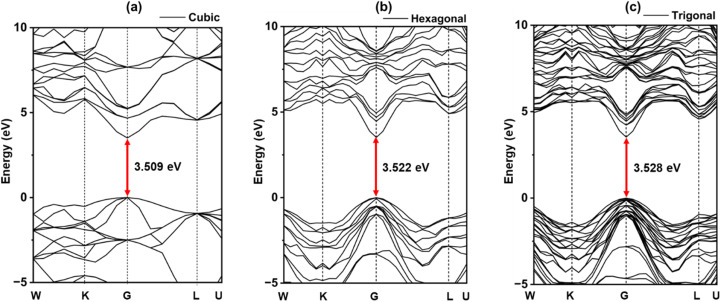
Band structure of ZnS polymorphs; (a) cubic, (b) hexagonal, (c) trigonal.

The band structure of ZnS polymorphs were calculated to compare their effects on the electronic structure. Fig. 2 shows the band structure located in the Brillouin region across the high-symmetry planes. The electrical band gap was computed by subtracting the valence band maximum from the conduction band minimum values. In this work, the band gaps of ZnS were found to be 3.51 eV, 3.52 eV, and 3.53 eV for cubic, hexagonal, and trigonal structures (Fig. 2a–c using the DFT + U approximation, closely matching the experimental values.^37^ Slightly higher band gap for hexagonal and trigonal is due to reduced orbital overlap in lower-symmetry structures, which raises the conduction band's energy.^38^ The valence band (VB) maximal and conduction band (CB) minimal values are found near the *Γ* point. There is no difference in the bandgap values between spin-up as well as spin-down pathways, confirming spin degeneracy. Minor variations in band dispersion along other high-symmetry points (*e.g.*, L or K) can influence optical absorption, effective mass, and carrier mobility. The slight increase in bandgap from cubic to trigonal polymorphs can be attributed to structural differences affecting orbital overlap and bonding interactions.

The motion of electrons produced by light determines the transport characteristics and the performance of the solar cell. Rapid charge transport mobility is preferred for a lower hole–electron duo reconciliation rate. The efficient mass of carriers of charges is negatively correlated with their motion.^39,40^1
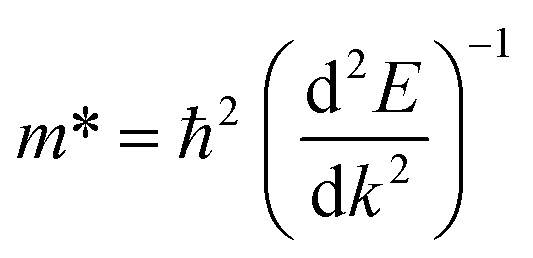
2
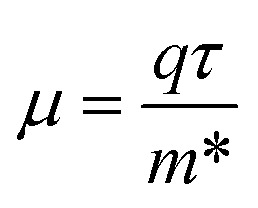
where m* is the effective mass, 
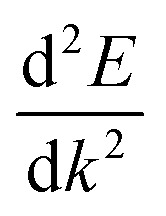
 is the value of the curving of bands close to the edge, *ħ* is the diminished Planck's constant, *µ* is the mobility of carriers, *τ* is the scattering time, and *q* is the carrier charge. By fitting the bands parabolically, the curve of the bands around the Gamma region was determined using OriginPro 2024 software (Copyright: OriginLab Corporation). The calculated carrier mobilities show that in the hexagonal phase the electron mobility is 343.2 cm^2^ V^−1^ s^−1^ and the mobility of hole is 92.6 cm^2^ V^−1^ s^−1^, in the trigonal phase the electron mobility is 334.4 cm^2^ V^−1^ s^−1^ and the mobility of hole is 77.2 cm^2^ V^−1^ s^−1^, while in the cubic phase the electron and hole mobilities are 262.2 cm^2^ V^−1^ s^−1^ and 45.2 cm^2^ V^−1^ s^−1^, correspondingly. Although such high mobility values are difficult to achieve, Hall effect measurements by Abdelali Talbi *et al.* reported that the ZnS film exhibits electron mobility of 500–800 cm^2^ (V^−1^ s^−1^) and a carrier concentration of approximately 1.235 × 10^17^ cm^−3^.^41^

### 3.2 Electronic density of states

**Fig. 3:**
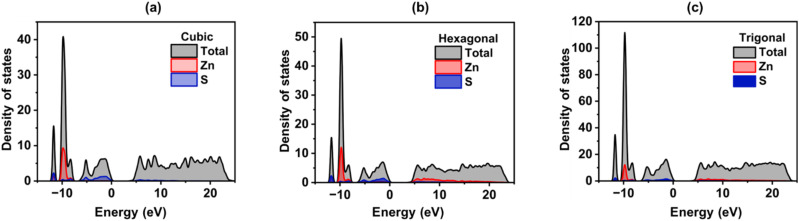
Density of states (DOS) and partial density of states (PDOS) of ZnS polymorphs; (a) cubic, (b) hexagonal, (c) trigonal.

Fig. 3 illustrates the total density of states, which is the sum of the partial DOS from sulfur and zinc in each of the three polymorphs. The Zinc 3d orbitals contribute significantly to the valence band, which is primarily composed of Sulfur 3p orbitals and lies below the Fermi level (0 eV). Zn-3d states contribute mostly to the bottom part of the band (–10 to –7 eV), whereas S-3p orbitals dominate the top part (–7 to 0 eV). Zn-4s/4p states are mostly composed of the conduction band, which is found above 0 eV, indicating that S-3p → Zn-4s/4p electronic excitations are the most common type. The main differences among the structures (Fig. 3a–c) are the shape of peak and energy allocation. The distinct stacking order and symmetry of the cubic (zinc blende), hexagonal (wurtzite), and trigonal structures alter the material's electronic properties. DFT + U (here, *U*_d_ = 8 eV and *U*_p_ = 4 eV) approach corrects the band gap close to the experimental value and correctly places the Zn-3d states. The electrical structures become more accurate, which is crucial for interpreting the optical and charge mobility characteristics of the material.

### 3.3 Optical properties

The wavelength range of 300–900 nm (or 1.38–4.13 eV) was purposefully chosen to encompass the UV, visible, and near-IR ranges in order to imitate the optical characteristics. This shows the material's great transparency in the visible and near-infrared areas, which is crucial for optoelectronic applications, and guarantees that the primary absorption edge of ZnS in the UV region is recorded.^42^

**Fig. 4:**
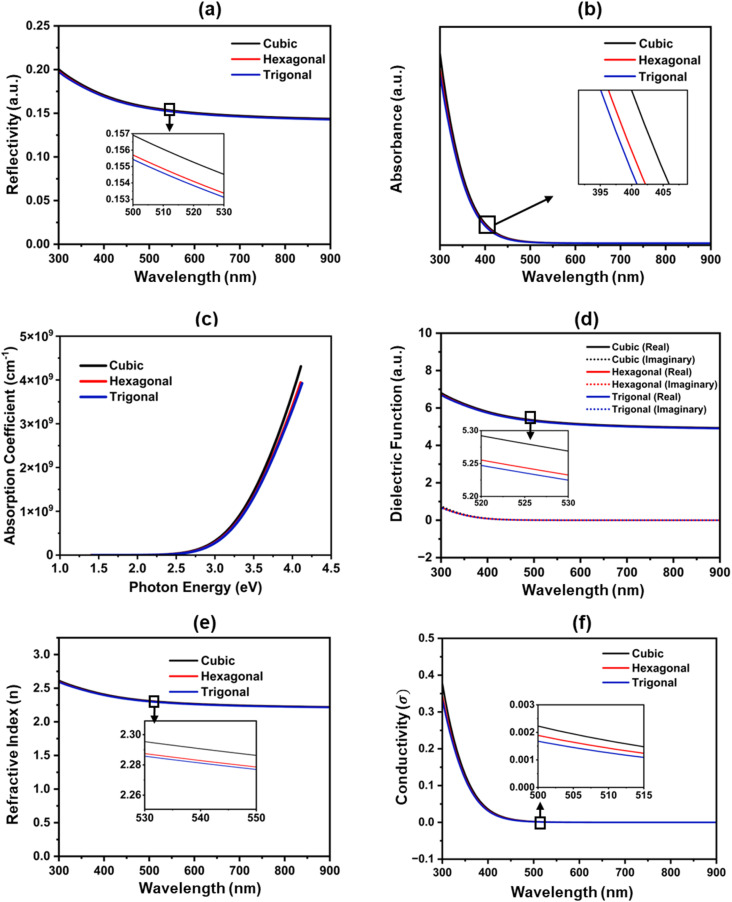
The optical functions of ZnS polymorphs; (a) reflectivity, (b) absorption, (c) absorption coefficient, (d) dielectric function, (e) refractive index, (f) conductivity.

The reflectivity of the three structures is shown in Fig. 4a. Reflectivity values are comparatively high at low photon energies and progressively decrease as energy rises. Cubic structure shows the highest reflectivity because of its higher refractive index and absorption properties. These results suggest that, relative to the other two stages, the cubic structure would exhibit somewhat more optical reflection in the UV-visible ranges. Fig. 4b illustrates the absorption spectra of the three polymorphs, where in the 395–405 nm region, a distinct absorption edge is seen. The ultraviolet spectrum (300–400 nm) exhibits strong absorption, but the visible and near-IR spectrums show a sharp reduction in absorption beyond 400 nm. This behaviour demonstrates the possibility of using ZnS as a window or BL in TFSCs and validates its great transparency in the visible spectrum^42,43^.

To investigate the optical characteristics of ZnS, the absorption coefficient (*α*) was computed using the following equation.^44^3
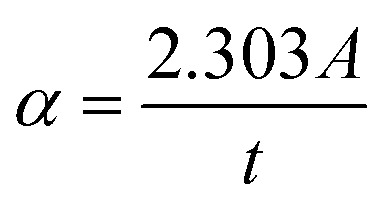
where *A* is the absorbance, *t* is the thickness of the film.^45^ As the materials will be used as buffer film in CZTSSe photovoltaic cells, the thickness is crucial. Generally, the BL thickness is taken at 50 nm to 100 nm. Here, 50 nm was selected to calculate the absorption coefficient of the layer. Absorption coefficient found between 10^4^ to 10^6^ cm^−1^ at 550 nm to 900 nm wavelength range (Fig. 4c), which matches with experimental data.^46,47^ The value absorption coefficient 10^8^ cm^−1^ or even 10^9^ cm^−1^ is also found, which is exceptionally high for a typical absorption coefficient and indicates a highly efficient light absorber in the UV range. Among the three structures, the cubic structure exhibits the highest absorption coefficient, indicating higher light absorption ability. Fig. 4d illustrates the dielectric function of real *ε*_1_(*ω*) and imaginary *ε*_2_(*ω*) at the wavelength region of 300–900 nm. The tangible and intangible components of the dielectric property eventually diminish with increasing wavelength and tend to stabilize in the visible-IR region. Among the three polymorphs, cubic ZnS exhibits the highest static dielectric constant value (≈5.3), followed closely by hexagonal ZnS (≈5.25), while trigonal ZnS shows the lowest value (≈5.2), as verified from the literature.^48^ This indicates that cubic ZnS has the strongest polarization capability and dielectric screening, which can effectively reduce exciton binding energy and enhance charge carrier separation, making it more suitable for optoelectronic applications. However, the small differences among the polymorphs also highlight that phase engineering can be used to fine-tune ZnS properties depending on whether stronger dielectric screening (cubic) or weaker exciton screening (trigonal) is desired.^49^ Refractive index (*n*) is also a significant factor for materials used in semiconductors. Fig. 4e depicts how the refractive index varies with wavelength (300–900 nm). It is found that the refractive index gradually decreases with increasing wavelength, indicating a normal dispersion behavior that is typical for wide band gap semiconductors.^50^ A closer comparison between the three phases reveals that the cubic structure exhibits the highest refractive index across the studied range, followed by the hexagonal and trigonal phases, with differences of about 0.01–0.03 between them, as highlighted in the inset figure. The cubic phase has a slightly higher refractive index due to its higher electronic polarizability and denser packing relative to the other phases. The gradual reduction of refractive index with wavelength is also consistent with the expected increase in film transmittance at longer wavelengths, as the refractive index is inversely proportional to transmittance. Overall, the results confirm that ZnS exhibits stable optical transparency in the visible-NIR region, with minor phase-dependent variations that may influence its suitability for optoelectronic applications.^51^Fig. 4f illustrates the optical conductivity (*σ*) of cubic, hexagonal, and trigonal phases as a function of wavelength. A significant peak arises near 400 nm due to interband electronic transitions, following which *σ* quickly falls and reaches a constant value at longer wavelengths.^52^ The inset figure shows that the cubic phase has greater conductivity than the hexagonal and trigonal phases, implying that the crystal structure has an impact on conductivity. While hexagonal ZnS possesses higher intrinsic carrier mobility than cubic ZnS, the cubic phase can still display higher bulk conductivity due to a greater carrier concentration and potentially improved film connectivity or reduced trap scattering. The electrical conductivity *σ* of a semiconductor is expressed as:

4 *σ* = *q*(*nµ*_*n*_ + *pµ*_*p*_)

where *q* is the elementary charge, *n* and *p* are the electron and hole concentrations, and *µ*_*n*_ and *µ*_*p*_ are the respective mobilities. This relation highlights that conductivity depends on both mobility and carrier density.

### 3.4 Device simulation

**Fig. 5:**
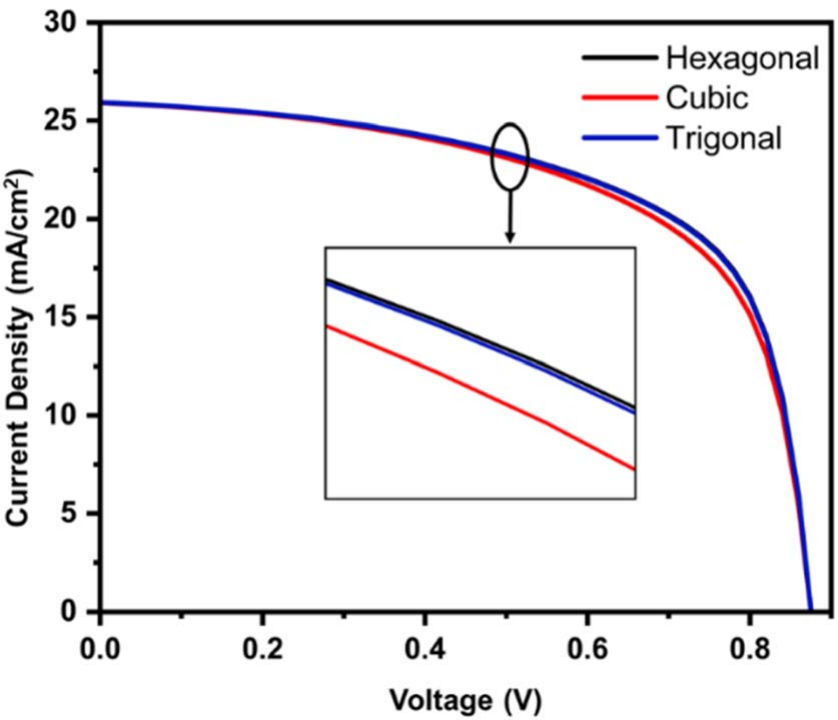
Comparative *J*–*V* curves for the structure ITO/AZO/ZnS/CZTSSe/Au w.r.t. ZnS polymorphs.

Fig. 5 depicts the electrical voltage–density (*J*–*V*) characteristics of CZTSSe solar cells that include ZnS polymorph layers of buffers in the ITO/AZO/ZnS/CZTSSe/Au design. The crystallographic phase of the ZnS BL appears to have an effect on the efficiency of the device. Solar cell with hexagonal ZnS exhibits superior photocurrent density (*J*_SC_ = 25.93 mA cm^−2^) and fill factor (FF = 62.5%), resulting in higher power conversion efficiencies (PCE = 14.18%) compared to the cubic phase (PCE = 13.74%) and Trigonal (PCE = 14.13%). Device simulations indicate that hexagonal ZnS delivers a higher PCE than cubic ZnS, implying that once a sufficient conductivity threshold is reached, device performance is dominated by interface band alignment, defect/trap recombination, and optical transparency rather than bulk conductivity. These results align with experimental and theoretical reports,^12,53,54^ confirming that ZnS polymorphs significantly influence the material's electrical properties. The relatively poor performance of the cubic ZnS buffer indicates the improper band alignment and enhanced interface recombination, whereas the hexagonal phase promotes more efficient carrier extraction. The highest electron mobility for hexagonal ZnS found from DFT + U calculations supports this performance. For the device optimization, further simulation will be conducted using a hexagonal ZnS structure.

### 3.5 Effect of thickness *vs.* donor density of ZnS layer

**Fig. 6:**
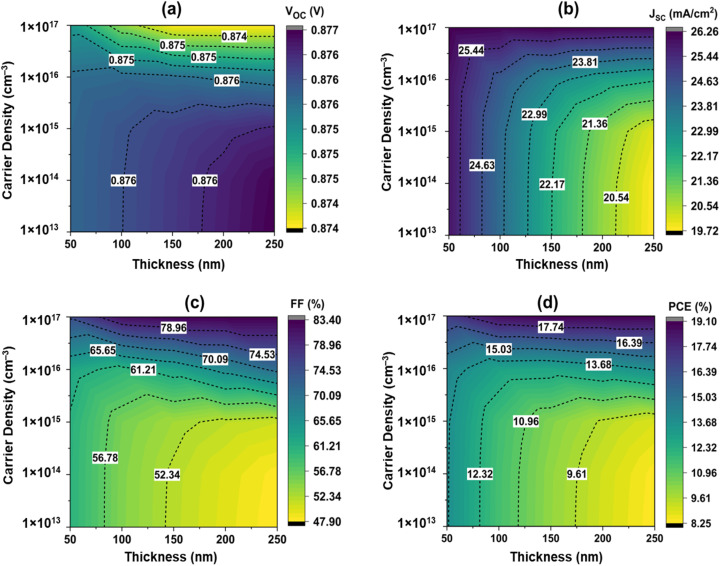
Variation in the solar cell properties with the thickness and carrier density of ZnS.

The ZnS BL shows a substantial impact on the PCE because it allows electrons to flow through to the window layer, reduces hole–electron reconciliation (*R*_e^−^h^+^_), and facilitates electron movement. To maximise PCE, the ZnS should have specified features, such as an optimum bandgap, appropriate thickness, increased mobility, and a greater carrier intensity. Overall, the degree of thickness of the material had a significant influence on carrier density. Fig. 6 demonstrates how adjustments in the thickness of the ZnS layer and the density of carriers affect the device's efficiency.

It is seen that while the thickness is kept consistent at 50 nm, carrier density rose from 1 × 10^13^ cm^−3^ to 1 × 10^17^ cm^−3^, there was an insignificant change in both *V*_OC_ and *J*_SC_, but an increment in FF from 60.7% to 75.1% and an increment in PCE from 13.75% to 17.25%. As the layer thickness of ZnS rose from 50 nm to 250 nm while keeping a steady carrier concentration of 1 × 10^13^ cm^−3^, there was a minor decrease in both *V*_OC_ and *J*_SC_, resulting in a fall in FF from 60.7% to 47.90% and a reduction in PCE from 13.75% to 8.29% (Fig. 6(a)–(d). With increasing thickness, the carriers generated in CZTSSe need to cross a thicker BL, which increases series resistance and reduces transport efficiency at the junction, resulting in reduced PCE.^55^ Finding the correct ZnS thickness is critical to achieving the maximum PCE. A ZnS thickness of 250 nm results in worse efficiency, probably because of the carrier lifespan linked with higher carrier density. The optimum thickness of ZnS was found at 150 nm and carrier density of 10^17^ cm^−3^ with PCE of 19.06%. A limited range close to 150 nm thickness was also studied, even though above mentioned thickness and carrier concentration offers the best efficiency. For thicknesses between 120 and 180 nm, the PCE is almost constant (19.01–19.07%), suggesting steady performance in this range. Since it provided the optimum balance between light absorption and carrier collection without raising series resistance, the 150 nm layer was chosen as the ideal one. In terms of doping, raising the donor concentration from 5 × 10^16^ to 2 × 10^17^ cm^−3^ enhances *J*_sc_ and FF and results in a higher PCE (19.54%), however raising it further to 3 × 10^17^ cm^−3^ somewhat reduces efficiency because of recombination. Therefore, a nearly ideal equilibrium between conductivity and recombination losses is offered by 1 × 10^17^ cm^−3^.

### 3.6 Effect of mobility and band gap of ZnS layer

**Fig. 7:**
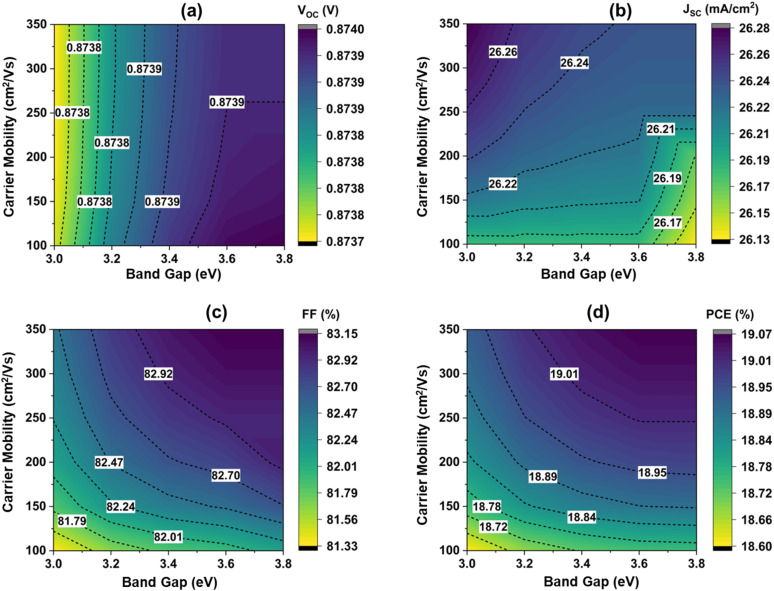
Variation in the solar cell properties with the band gap and carrier mobility of ZnS.

Carrier mobility is a critical factor in device performance because it explains how rapidly electrons and holes may flow across the semiconductor over an electric field that is applied.^56,57^Fig. 7 illustrates the effect of the band gap of the ZnS BL and carrier mobility on device performance. The band gap was varied 3–3.8 eV, and the carrier mobility varied 100 to 350 cm^2^/*vs.*, aligning with experimentally found data.^58–62^ It is seen that when the bandgap is constant at 3.6 eV, carrier mobility varied, there was a practically insignificant change in *V*_OC_, a slight increment in *J*_SC_ from 26.18 to 26.24 mA cm^−2^, and FF from 81.92% to 83.15% which increases PCE from 18.75% to 19.06%. The mobility improved the fill factor (FF), as higher carrier mobility reduces resistive losses, recombination rates, and enhances device performance as well as stability.^63^ When the bandgap varies from 3 to 3.8 eV, carrier mobility constant at 100 cm^2^ V^−1^ s^−1^, there was a practically insignificant change in *V*_OC_, and *J*_SC_ slightly decreased, resulting in a slight increment in FF from 81.3% to 82.1% resulting in an increment in PCE from 18.6% to 18.75%. Since ZnS has a wide bandgap (>3 eV), it does not directly limit *V*_OC_ and provides good band alignment with CZTSSe. The *J*_SC_ decreases with increasing band gap due to the reduced absorption of longer-wavelength photons, but higher mobility helps sustain *J*_SC_ by enabling more efficient charge collection.^64^ The optimized band alignment due to the combination of wide bandgap ZnS and high mobility reduces series resistance, contributing to a higher FF and enhancing device efficiency.

It is seen that when the bandgap is constant at 3.6 eV, carrier mobility varied, there was a practically insignificant change in *V*_OC_, a slight increment in *J*_SC_ from 26.18 to 26.24 mA cm^−2^, and FF from 81.92% to 83.15% which increases PCE from 18.75% to 19.06%. The mobility improved the fill factor (FF), as higher carrier mobility reduces resistive losses, recombination rates, and enhances device performance as well as stability.^63^ When the bandgap varies from 3 to 3.8 eV, carrier mobility constant at 100 cm^2^ V^−1^ s^−1^, there was a practically insignificant change in *V*_OC_, and *J*_SC_ slightly decreased, resulting in a slight increment in FF from 81.3% to 82.1% resulting in an increment in PCE from 18.6% to 18.75%. Since ZnS has a wide bandgap (>3 eV), it does not directly limit *V*_OC_ and provides good band alignment with CZTSSe. The *J*_SC_ decreases with increasing band gap due to the reduced absorption of longer-wavelength photons, but higher mobility helps sustain *J*_SC_ by enabling more efficient charge collection.^64^ The optimized band alignment due to the combination of wide bandgap ZnS and high mobility reduces series resistance, contributing to a higher FF and enhancing device efficiency.

### 3.7 Effect of interface defect

**Fig. 8:**
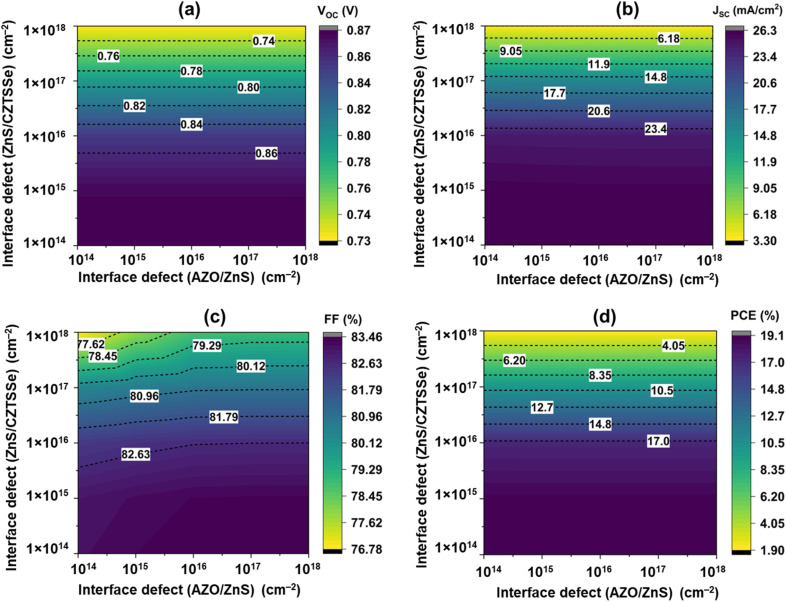
Variation in the solar cell parameters with interface defects at the CZTSSe/ZnS and AZO/ZnS junctions.

Interface defects at the CZTSSe/ZnS and AZO/ZnS junctions arise from lattice and chemical mismatches, creating trap states that accelerate the Shockley–Read–Hall recombination.^65^ These defects reduce carrier lifetime and hinder charge extraction, lowering *V*_OC_, *J*_SC_, and FF unless effectively passivated.^66^Fig. 8a–d depicts the influence of interface defects on the photovoltaic characteristics of the examined solar cell structure, emphasising the interdependent effect of interface defect concentrations at the AZO/ZnS and ZnS/CZTSSe interfaces on cell efficiency.

Keeping the AZO/ZnS interface defect density is fixed at 1 × 10^14^ cm^−2^, increasing the ZnS/CZTSSe defect density from 1 × 10^14^ to 1 × 10^18^ cm^−2^, *V*_OC_ reduces from 0.873 V to 0.726 V, *J*_SC_ from 26.24 mA cm^−2^ to 3.484 mA cm^−2^, and PCE from 19.07% to 1.94%, while FF decreased from 83.149% to 76.79%. This shows that *J*_SC_ are highly sensitive to ZnS/CZTSSe defects. Maximum *J*_SC_ values are observed under low defect density at 1 × 10^14^ cm^−2^, while higher defect densities lead to significant carrier losses due to interface recombination, thereby drastically reducing *J*_SC_. The *V*_OC_ reduction is due to enhanced non-radiative recombination caused by interface trap states, which increase the recombination current.^67,68^ As a result of enhanced recombination and a weakened built-in electric field that hampers charge extraction, the series resistance (*R*_s_) increases while the shunt resistance (*R*_sh_) decreases, ultimately causing a decline in the FF. When the ZnS/CZTSSe interface defect density exceeds 1 × 10^16^ cm^−3^, a drastic decrease in device performance is observed. Various types of defects at this junction act as recombination centers, significantly hindering charge transport and reducing photovoltaic efficiency. Previous studies provide important insights into the origin and effects of such defects at the ZnS/CZTSSe interface. For example, De Oliveira *et al.* reported that Zn vacancies are more likely to form under sulfur-rich and zinc-poor conditions, leading to sub-bandgap absorption and altered electronic properties.^69^ Similarly, Hoang *et al.* found that both cation and anion vacancies, as well as antisite defects, can introduce deep electronic states that behave as donors or acceptors depending on the Fermi level position.^70^ These findings suggest that the high interface defect density (≥10^16^ cm^−3^) likely originates from such intrinsic point defects, which act as recombination centers. Therefore, appropriate interface engineering or process optimization—such as controlling sulfurization conditions and Zn chemical potential—can effectively mitigate their detrimental effects and improve device performance. When the ZnS/CZTSSe defect density is fixed at 1 × 10^15^ cm^−2^ and the AZO/ZnS defect density increases from 1 × 10^14^ to 1 × 10^18^ cm^−2^, there is no significant impact on photovoltaic performance. A slight increase in FF with increasing AZO/ZnS interface defect density likely arises from minor adjustments in the junction potential or charge transport balance at the transparent conducting interface. AZO/ZnS interface lies in the transparent conducting region to transport photoelectron, where carrier generation is negligible; hence, defects did not significantly affect charge transportation.

### 3.8 Effect of bulk defect density of ZnS layer

**Fig. 9:**
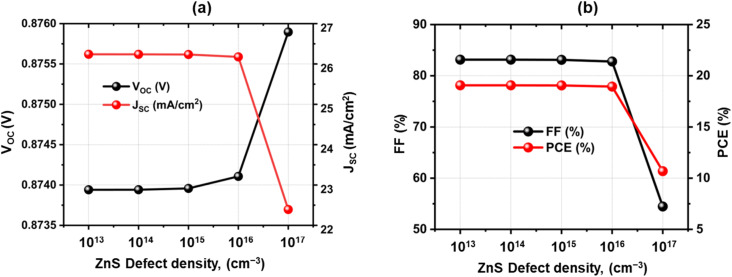
Variation in the solar cell parameters with bulk defects of ZnS.

Defects are usually located in the bulk materials or at the contact point.^71^ Higher concentrations of defects in bulk result in a higher rate of recombination along with a decreased strength, a quicker rate of film breakdown, and a decline in the device's effectiveness.^72^Fig. 8(a and b) illustrate the influence of defect density of ZnS BL on photovoltaic cell performance has been investigated. The variation of *V*_OC_, *J*_SC_, FF, and PCE are shown for ZnS defect concentrations between 1 × 10^13^ and 1 × 10^17^ cm^−3^. D. Kurbatov *et al.* reported that ZnS films exhibit trap densities in the range of 5 × 10^14^ to 1.5 × 10^15^ cm^−3^.^73^ The defect densities used in this simulation was chosen to align with these experimental values. It is shown that the photovoltaic performance barely changes when the defect density is increased from 1 × 10^13^ to 1 × 10^16^ cm^−3^. Since ZnS mainly acts as a wide-bandgap BL, and carriers generated in CZTSSe can still transfer efficiently to the TCO. After 10^16^ cm^−3^, there is a noticeable drop in *J*_SC_, FF, and PCE and an increase in *V*_OC_. This phenomenon arises from the higher defect density, which enhances the recombination current and accelerates carrier recombination. Finally, at the highest possible value of 10^17^ cm^−3^, the efficiency drops from 19.07% to the minimal effectiveness of 10.7%. Because the carriers created by light recombine inside the BL prior to reaching the electrode, *J*_SC_ drastically drops at large bulk defect densities in ZnS.^74^ As recombination increases, the FF also declines, because the bulk defects increase series resistance and hinder efficient charge extraction.^75^ Consequently, PCE drops drastically. Interestingly, *V*_OC_ shows an unusual incremental trend under these conditions. This occurs because excessive recombination alters the quasi-Fermi level splitting and artificially raises *V*_OC_.^76^ However, this increase does not reflect the performance improvement, as the overall device power output collapses due to severe recombination losses (Fig. 9).

### 3.9 Effect of temperature on device performance

**Fig. 10:**
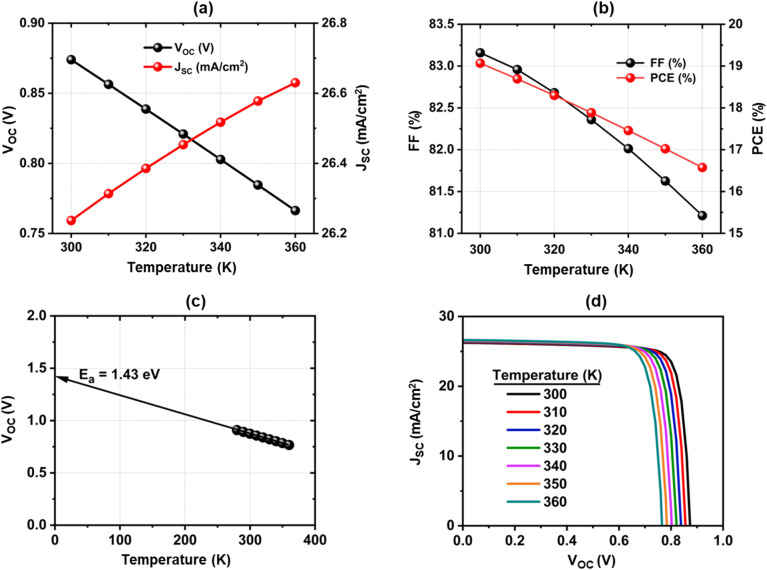
Effect of temperature on device performance; (a) *V*_OC_*vs. J*_SC_, (b) FF *vs.* PCE, (c) Determination of activation energy of recombination, (d) *J*–*V* curve of the proposed device.

The effectiveness of photovoltaic cells is greatly impacted by temperature. Perovskite solar cells typically operate at temperatures between 300 and 325 K in atmospheric settings. As shown in Fig. 10a and b, the device parameters vary with operating temperature: *V*_OC_, FF, and PCE exhibit a linear decline, while *J*_SC_ increases. Higher carrier recombination results in an increase in reverse saturation current, which lowers the *V*_OC_ and causes this phenomenon.^77^*J*_SC_ increases with temperature mainly because of bandgap narrowing (eqn (4)), which enhances light absorption and carrier generation. This effect causes a slight redshift the absorption edge and enhances light absorption in the longer wavelength region (near the band-edge region of 750–900 nm).^78^Eqn (5) indicates that a smaller *E*_g_ allows easier thermal excitation of electrons, so with higher *T* and lower *E*_g_, more electron–hole pairs are generated. At the same time carrier recombination increases at higher temperatures. But the reduced bandgap and higher intrinsic carrier concentration (*n*_i_) enhance carrier generation.

Moreover, a rise in cell temperature influences material conductivity by enhancing charge carrier scattering and recombination, which in turn reduces both the FF and PCE.^79^Fig. 10d presents the *J*–*V* curves at different temperatures, showing that the reduction in PCE with rising temperature occurs at an almost constant rate. The energy of activation of recombination is depicted in Fig. 10c and is associated with the subsequent equations [80]:5
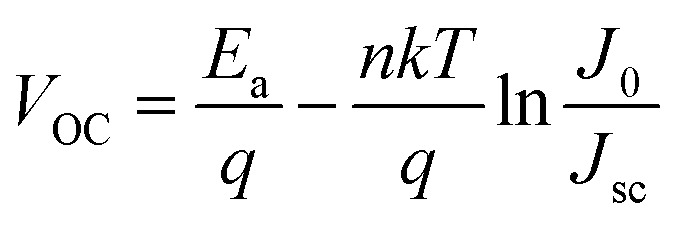
6
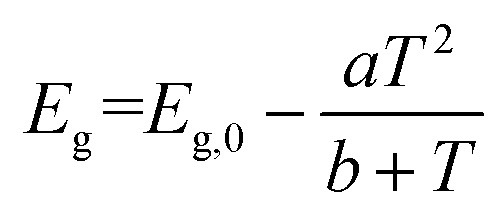
7
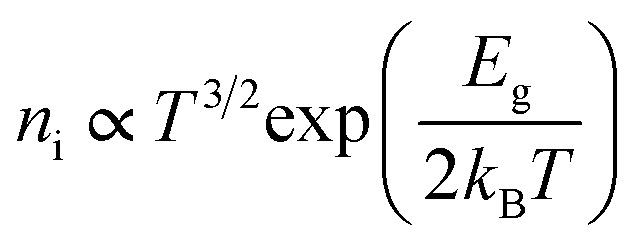


where *E*_a_ is the activating energy of recombination, *kT*/*q* is the thermal voltage, *J*_0_ is the weakened temperature-dependent preliminary factor, and *n* and *J*_0_ are the factor of ideality and saturation current density of the diode, respectively, *E*_g,0_ is the band gap at *T* = 0 K, and a and *b* are constants, *E*_g_ is the bandgap, *k*_B_ is Boltzmann's constant, and *T* is temperature. Energy cliffs [CBO (−)] and spikes [CBO (+)] arise when the electron affinities of the ETL and absorber differ. If the ETL conduction band lies below that of the absorber, a cliff-type CBO (−) forms at the ETL/absorber interface, offering no electron barrier. In this case, interface recombination dominates when the recombination activation energy (*E*_a_) is less than the absorber's bandgap. Such cliff-type alignment reduces resistance to electron transfer, decreases *E*_a_, and negatively impacts *V*_OC_, *J*_SC_, FF, and overall PCE. In the present case, the slightly higher *E*_a_ (1.36 eV) compared to the bandgap (1.3 eV) is attributed to this cliff-type band alignment but is negligible and effectively treated as coinciding with *E*_g_. Shockley–Read–Hall (SRH) recombination occurs at the bulk density or in the absorber's charged space region if *E*_a_ and *E*_g_ overlap. If *E*_a_ < *E*_g_: interface or defect-related recombination dominates. The likelihood of recombination increases with increasing the gap between *E*_g_ and *E*_a_.

### 3.10 Effect of back contact work function

**Fig. 11:**
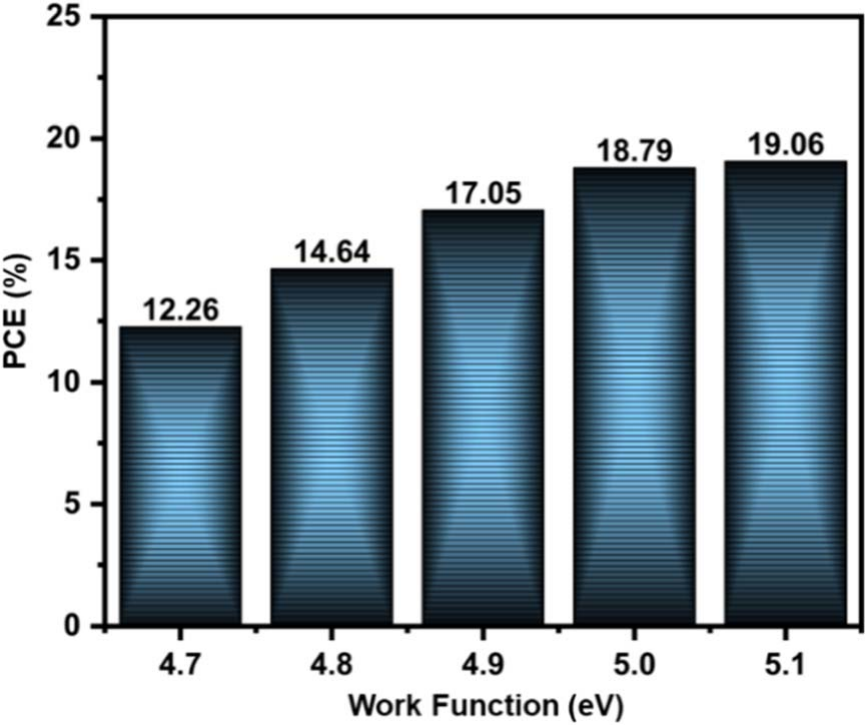
Effect of back contact electrodes on device performance.

One of the most important factors influencing PSC performance is the back electrode work function. An increase in the work function reduces the barrier height at the contact, leading to stronger ohmic interaction and improved charge extraction. To examine this effect, simulations were carried out using different metals with varying work functions: Ag (4.7 eV), Fe (4.8 eV), Cu (4.9 eV), C (5.0 eV), and Au (5.1 eV), as shown in Fig. 11. The findings show that devices with higher work function electrodes exhibit significantly enhanced PCE, reaching a maximum of ∼19% at 5.1 eV. In contrast, lower work function metals form Schottky-type contacts, which hinder charge transport and reduce efficiency. Recombination losses and hole extraction are controlled by the majority carrier barrier height, which is influenced by the back-contact metal. As the metal work function increases, the barrier height lowers. For example, the majority carrier barrier heights in relation to the valence band are around 0.73, 0.63, 0.53, 0.43, and 0.33 eV for work function values of 4.7, 4.8, 4.9, 5.0, and 5.1 eV, respectively. This demonstrates that Au, which has the higher work function (5.1 eV), raises the FF and PCE by lowering the barrier for effective hole extraction, and decreasing recombination. This trend highlights the importance of selecting an electrode with a high work function to ensure effective band alignment and minimize energy loss. Among the simulated materials, Au demonstrates the higher efficiency because of its high work function, while Ag shows the lowest due to its lower work function and associated Schottky barrier formation.

## 4 Conclusion

In this study, first-principles DFT + U calculations were employed to investigate the structural, electronic, and optical properties of ZnS in its cubic, hexagonal, and trigonal phases. The calculated band gaps of 3.51, 3.52, and 3.53 eV for cubic, hexagonal, and trigonal ZnS, respectively, show excellent agreement with reported experimental values. Carrier mobilities calculated from band structure revealed that the hexagonal phase exhibited the highest electron (343.2 cm^2^ V^−1^ s^−1^) and hole (92.6 cm^2^ V^−1^ s^−1^) mobilities, followed by the trigonal and cubic phases, suggesting superior charge transport in the wurtzite structure. The density of states analysis further demonstrated that the Zn-3d orbitals dominate the lower valence band, while the S-3p orbitals govern the upper valence band and Zn-4s/4p states form the conduction band, consistent with S-3p → Zn-4s/4p electronic excitations. Device simulations with ZnS BL further showed that the hexagonal phase enables the best photovoltaic performance, with *J*_SC_ (25.93 mA cm^−2^), FF (62.5%), and PCE (14.18%) compared to the cubic (PCE 13.74%) and trigonal (PCE 14.13%) phases. The enhanced efficiency of hexagonal ZnS arises from favorable band alignment and reduced interface recombination. Optimized conditions yielded a maximum PCE of 19.06% (*V*_oc_ = 0.87 V, *J*_SC_ = 26.23 mA cm^−2^, FF = 83.14%). Overall, these findings highlight the role of ZnS polymorph selection in tailoring interfacial properties and achieving high-performance CZTSSe solar cells. The insights gained from this work provide a valuable guideline for experimental efforts to integrate phase-engineered ZnS as an effective buffer layer in sustainable photovoltaic devices.

## Author contributions

Md. Azad Patwary: conceptualization, writing – original draft; Aqib Adnan Shafin: writing and editing; Md. Morshed Alam: review & editing; Rajat Kumar Singh Durjoy: editing & visualization; Norasikin Ahmad Ludin: software, editing & formal analysis; Mohd Sukor Su'ait: review & editing; Md. Akhtaruzzaman: editing and formal analysis; M. Mottakin: supervision & writing – review & editing.

## Conflicts of interest

There are no conflicts to declare.

## Data Availability

Data will be made available upon a reasonable request to the corresponding author.

## References

[cit1] Al-Ali S., Olabi A. G., Mahmoud M. (2025). Energy Convers. Manage.:X.

[cit2] Xia Y., Chu X., Jiang Y., Du Y., Zhao W., Liu Y., Sui Y., Yao B., Liu Y. (2025). Chem. Eng. J..

[cit3] Laghchim E., Raidou A., Fahmi A., Fahoume M. (2022). Micro Nanostruct..

[cit4] Ali M. H., Haque M. D., Hossain M. F., Islam A. Z. M. T. (2025). RSC Adv..

[cit5] Sivasankar S. M., Amorim C. de O., da Cunha A. F. (2025). J. Compos. Sci..

[cit6] Ngqoloda S. , Ngwenya T., Raphulu M., Ngqoloda S., Ngwenya T. and Raphulu M., DOI:10.5772/INTECHOPEN.1008691

[cit7] Agrawal D., Patel S. L., Himanshu, Chander S., Dhaka M. S. (2020). Opt. Mater..

[cit8] Kaushalya S. L. P., Purohit A., Chander S., Dhaka M. S. (2018). Phys. E.

[cit9] Ali Md. H., Hossain Md. F., Hossain Md. M., Haque Md. D., Islam A. Z. Md. T. (2024). Opt. Quant. Electron..

[cit10] Sikder S., Hasan M. K., Mamur H., Bhuiyan M. R. A. (2025). Hybrid Adv..

[cit11] Chelvanathan P., Yusoff Y., Haque F., Akhtaruzzaman M., Alam M. M., Alothman Z. A., Rashid M. J., Sopian K., Amin N. (2015). Appl. Surf. Sci..

[cit12] Arsad A. Z., Zuhdi A. W. M., Abdullah S. F. (2025). Ceram. Int..

[cit13] Zagorac D., Zagorac J., Pejić M., Matović B., Schön J. C. (2022). Nanomaterials.

[cit14] Dou B., Li Y., Fang S., Zhao Q., Cheng H., Liang P., Wang L. (2024). Adv. Photonics Res..

[cit15] Almotiri R. A. (2025). J. Mater. Sci.: Mater. Electron..

[cit16] Balnan İ., Horoz S., Kaya K. K., Orak C. (2025). J. Australas. Ceram. Soc..

[cit17] Kamruzzaman M., Mia M. S. Z., Hossain M. F., Tanveer Karim A. M. M., Afrose R., Khan M. K. R. (2025). Mater. Sci. Eng. B.

[cit18] La Porta F. A., Gracia L., Andrés J., Sambrano J. R., Varela J. A., Longo E. (2014). J. Am. Ceram. Soc..

[cit19] Nguyen M., Ernits K., Tai K. F., Ng C. F., Pramana S. S., Sasangka W. A., Batabyal S. K., Holopainen T., Meissner D., Neisser A., Wong L. H. (2015). Sol. Energy.

[cit20] Park J. Y., Chalapathy R. B. V., Lokhande A. C., Hong C. W., Kim J. H. (2017). J. Alloys Compd..

[cit21] Vallisree S., Thangavel R., Lenka T. R. (2018). J. Mater. Sci.: Mater. Electron..

[cit22] Hirwani L., Chandrakar V., Yadu G., Oudhia A., Verma B., Ramadhin (2025). Res. Text Theory.

[cit23] Vallisree S., Ghosh A., Thangavel R., Lenka T. R. (2018). J. Mater. Sci.: Mater. Electron..

[cit24] Adnan M., Wang Q., Sohu N., Du S., He H., Peng Z., Liu Z., Zhang X., Bai C. (2023). Materials.

[cit25] Luo Y., Sun W., Han H., Peng J., Jiang F. (2025). Int. J. Min. Sci. Technol..

[cit26] Sharma M., Mishra D., Kumar J. (2019). Phys. Rev. B.

[cit27] Liu B., Su W. S., Wu B. R. (2022). RSC Adv..

[cit28] Ghaleb A. M., Ahmed A. Q. (2022). Chalcogenide Lett..

[cit29] Sanad M. F., Shalan A. E., Ahmed M. A., Messih M. F. A. (2021). RSC Adv..

[cit30] Panda S., Patnaik P. (2025). Bulg. J. Phys..

[cit31] Ghaleb A. M. , Benkrima Y., Ahmed A. Q. and Ghaleb Z. T., Problems of Atomic Science and Technology, 2024, 103–109

[cit32] Srivastava A., Dua P., Lenka T. R., Tripathy S. K. (2021). Mater. Today Proc..

[cit33] Jhuma F. A., Shaily M. Z., Rashid M. J. (2019). Mater. Renew. Sustain. Energy.

[cit34] Zepeda Medina J. C., Rosendo Andrés E., Morales Ruíz C., Camacho Espinosa E., Treviño Yarce L., Galeazzi Isasmendi R., Romano Trujillo R., García Salgado G., Coyopol Solis A., Nieto Caballero F. G., Carranza Sanchez A. C. (2023). Heliyon.

[cit35] Sultan Md. Z., Shahriar A., Tota R., Howlader Md. N., Rodro H. H., Akhy M. J., Al Rashik Md. A., Sultan Md. Z., Shahriar A., Tota R., Howlader Md. N., Rodro H. H., Akhy M. J., Al Rashik Md. A. (2024). Energy Power Eng..

[cit36] Sadanand, Dwivedi D. K. (2019). Sol. Energy.

[cit37] Oliva A. I. , González-Chan I., Rejón V., Rojas J., Patiño R. and Aguilar D., Program and Abstract Book - 2010 7th International Conference on Electrical Engineering, Computing Science and Automatic Control, CCE 2010, 500–503

[cit38] Sabino F. P., Zhao X. G., Dalpian G. M., Zunger A. (2024). Phys. Rev. B.

[cit39] Nolan M., Elliott S. D. (2006). Phys. Chem. Chem. Phys..

[cit40] Zhang J., Zhou P., Liu J., Yu J. (2014). Phys. Chem. Chem. Phys..

[cit41] Rodrigues C. G. (2006). Microelectronics J..

[cit42] Vishwakarma R. (2017). Ukrainian J. Phys..

[cit43] Kucukarslan A., Kus E. (2021). Int. j. sci. res. sci. technol..

[cit44] Kozhevnikova N. S., Melkozerova M. A., Enyashin A. N., Tyutyunnik A. P., Pasechnik L. A., Baklanova I. V., Suntsov A. Y., Yushkov A. A., Buldakova L. Y., Yanchenko M. Y. (2022). J. Phys. Chem. Solids.

[cit45] Yadav P., SanthiBhushan B., Srivastava A. (2025). Beilstein J. Nanotechnol..

[cit46] Arif S. (2025). RSC Adv..

[cit47] Vivas M. G., Cury J. F., Schiavon M. A., Mendonca C. R. (2013). J. Phys. Chem. C.

[cit48] Göde F., Güneri E., Kariper A., Ulutaş C., Kirmizigül F., Gümüş C. (2011). J. Phys.:Conf. Ser..

[cit49] Ong H. C., Chang R. P. H. (2001). Appl. Phys. Lett..

[cit50] Adewale A. A., Chik A., Adam T., Joshua T. M., Durowoju M. O. (2021). Mater. Today Commun..

[cit51] Abd-Elrahman M. I., Hafiz M. M., Abdelraheem A. M., Abu-Sehly A. A. (2015). Opt. Mater..

[cit52] Sabitha C., Joe I. H., Kumar K. D. A., Valanarasu S. (2018). Opt. Quant. Electron..

[cit53] Lashgari H., Boochani A., Shekaari A., Solaymani S., Sartipi E., Mendi R. T. (2016). Appl. Surf. Sci..

[cit54] Simya O. K., Mahaboobbatcha A., Balachander K. (2015). Superlattices Microstruct..

[cit55] Arya S., Mahajan P., Gupta R., Srivastava R., kumar Tailor N., Satapathi S., Sumathi R. R., Datt R., Gupta V. Prog. Solid State Chem..

[cit56] Kumar A., Ranjan P. (2021). Sol. Energy.

[cit57] Choi H., Lee N., Park H., Choi Y., Yuk H., Lee J., Lee S. G., Lee E. J., Jeon H. (2021). Optik (Stuttg).

[cit58] Talbi A., Khaaissa Y., Mansouri F., El Khouja O., Chaouiki A., Nouneh K., AlObaid A. A. (2025). ACS Omega.

[cit59] Habib K. M. (2015). Univ. Aden J. Nat. Appl. Sci..

[cit60] Kumar P., Maikap S., Singh K., Kapoor A., Kumar S., Kumar N., Thi Quynh Hoa T., Van Vu L., Dinh Canh T., Ngoc Long N. (2009). J. Phys.:Conf. Ser..

[cit61] Uzar N., Arikan M. C. (2011). Bull. Mater. Sci..

[cit62] Dakua P. K., Dash R. K., Laidouci A., Bhattarai S., Dudekula U., Kashyap S., Agarwal V., Rashed A. N. Z. (2024). J. Electron. Mater..

[cit63] Khaled Mostaque S., Kumar Mondal B., Hossain J. (2022). Results in Optics.

[cit64] Varley J. B., Lordi V. (2014). J. Appl. Phys..

[cit65] Huang T. J., Yin X., Qi G., Gong H. (2014). Phys. Status Solidi RRL.

[cit66] Peng Z., Wortmann J., Hong J., Zhou S., Bornschlegl A. J., Haffner-Schirmer J., Corre V. M. L., Heumüller T., Osvet A., Rand B. P., Lüer L., Brabec C. J. (2025). Adv. Energy Mater..

[cit67] Liu Q., Vandewal K. (2023). Adv. Mater..

[cit68] Fan R., Zhou W., Huang Z., Zhou H. (2020). EnergyChem.

[cit69] Abbas S., Bajgai S., Chowdhury S., Najm A. S., Jamal M. S., Techato K., Channumsin S., Sreesawet S., Channumsin M., Laref A., Rahman K. S., Holi A. M. (2022). Materials.

[cit70] Kesavan A. V., Rao A. D., Ramamurthy P. C. (2017). ACS Appl. Mater. Interfaces.

[cit71] Lukyanchikova N. B., Pekar G. S., Tkachenko N. N., Shin H. Mi, Sheinkman M. K. (1977). Solid State Electron.

[cit72] Salman M. U., Mehak M., Ali U., Din G. M. U., Ramay S. M., Younis M., Atiq S. (2025). RSC Adv..

[cit73] Lin P., Lin L., Yu J., Cheng S., Lu P., Zheng Q. (2014). J. Eng. Appl. Sci..

[cit74] Sobayel K., Rahman K. S., Karim M. R., Aijaz M. O., Dar M. A., Shar M. A., Misran H., Amin N. (2018). Chalcogenide Lett..

[cit75] Bin Rafiq K. S., Mottakin M., Muhammad G., Techato K., Sopian K., Akhtaruzzaman M. (2022). Jpn. J. Appl. Phys..

[cit76] Hossain M. K., Arnab A. A., Das R. C., Hossain K. M., Rubel M. H. K., Rahman M. F., Bencherif H., Emetere M. E., Mohammed M. K. A., Pandey R. (2022). RSC Adv..

[cit77] Wang K., Gunawan O., Todorov T., Shin B., Chey S. J., Bojarczuk N. A., Mitzi D., Guha S. (2010). Appl. Phys. Lett..

[cit78] Yang K. J., Sim J. H., Son D. H., Jeon D. H., Hwang D. K., Nam D., Cheong H., Kim S. Y., Kim J. H., Kim D. H., Kang J. K. (2017). J. Ind. Eng. Chem..

[cit79] Mottakin M., Sarkar D. K., Selvanathan V., Rashid M. J., Sobayel K., Hasan A. K. M., Ariful Islam M., Muhammad G., Shahiduzzaman M., Akhtaruzzaman M. (2023). Optik (Stuttg).

